# Enhanced Risk Stratification for Sentinel Lymph Node Biopsy in Head and Neck Melanoma Using the Merlin Assay (CP-GEP)

**DOI:** 10.1245/s10434-024-16551-8

**Published:** 2024-11-23

**Authors:** Ani Pazhava, Wesley Y. Yu, Frank Z. Jing, Sheena Hill, Bethany R. Rohr, Kord S. Honda, Félicia Tjien-Fooh, Renske Wever, Jvalini Dwarkasing, Tina J. Hieken, Alexander Meves

**Affiliations:** 1 Department of Dermatology, Mayo Clinic, Rochester, MN USA; 2 Department of Dermatology, Oregon Health and Science University, Portland, OR USA; 3 Department of Dermatology, University Hospitals Cleveland Medical Center, Cleveland, OH USA; 4 SkylineDx B.V., Rotterdam, The Netherlands; 5 Department of Surgery, Mayo Clinic, Rochester, MN USA

## Abstract

**Background:**

Sentinel lymph node biopsy (SLNB) for head and neck melanomas involves complex challenges due to intricate lymphatic networks and delicate anatomic structures. The Merlin Assay (CP-GEP), merging clinicopathologic data with gene expression profiling, offers a non-invasive method to identify patients who have a low risk for nodal metastasis, potentially sparing these low-risk patients from surgical procedures.

**Methods:**

This study evaluated 250 clinically node-negative patients with stage I, II, or III melanoma from the Mayo Clinic and University Hospitals Cleveland Medical Center who had tumors in the head and neck region diagnosed between 2004 and 2021. All the patients underwent SLNB. The Merlin Assay, using the CP-GEP model, combines patient age at diagnosis, Breslow thickness, and gene expression of eight specific genes from the primary tumor to predict the risk of nodal metastasis.

**Results:**

The SLNB positivity rate was 14% overall, and CP-GEP predicted a possible 40.8% reduction in SLNB procedures with a negative predictive value (NPV) of 98%. For 215 SLNB-negative patients (5-year recurrence-free survival [RFS] of 76.9%, distant metastasis-free survival [DMFS] of 84.3%, and melanoma-specific survival [MSS] of 90.6%), CP-GEP improved risk stratification by identifying 100 patients as low risk with 5-year RFS of 86.1%, DMFS of 92.7%, and MSS of 95.3%. Among 167 T1–T2 patients, the SLNB positivity rate was 8.4%, and CP-GEP achieved an SLNB reduction rate of 56.3% with an NPV of 98.9%.

**Conclusions:**

The Merlin Assay effectively categorizes head and neck melanoma patients by risk, enabling more accurate clinical decision-making regarding SLNB and follow-up evaluation, especially for early-stage melanoma patients.

**Supplementary Information:**

The online version contains supplementary material available at 10.1245/s10434-024-16551-8.

Sentinel lymph node biopsy (SLNB) is the gold standard for staging clinically node-negative cutaneous melanoma patients and guiding adjuvant therapy options.^1^ According to the Multicenter Selective Lymphadenectomy Trial (MSLT-I), excising sentinel lymph nodes (SLNs) with micrometastases provides regional disease control. However, removing SLNs without metastases is not therapeutically beneficial.^2^ This highlights the crucial challenge of precise patient selection for SLNB. Melanomas of the head and neck are especially challenging in their clinical evaluation.^3^ The complex lymphatic drainage in this region complicates the identification of SLNs, and the presence of vital structures adds to the task.^4,5^ Assessment of SLN status in the head and neck region has shown a higher likelihood of false-negative results, leading to an underestimation of prognosis regarding the risk of disease recurrence.^6^ Tools that improve risk stratification over the current standard of care (i.e., histopathology of the primary tumor and SLNs) are therefore needed.

The Merlin Assay, a non-invasive test comprising a prediction model that combines clinicopathologic (CP) variables with the gene expression-profiling (GEP) of diagnostic biopsy tissue,^7^ has previously been developed. It is intended to identify primary cutaneous melanoma patients at low risk for nodal metastases who may therefore forgo SLNB. The assay has been validated in both European and U.S. patient cohorts, demonstrating its ability to predict the likelihood of nodal metastases^8–11^ and the risk of disease recurrence.^12–14^ This study assessed the performance of CP-GEP to predict nodal metastasis and disease recurrence specifically for patients with melanoma of the head and neck area.

## Methods

### Study Population

Our cohort for analysis consisted of 250 clinically node-negative patients who had stage I, II, or III primary cutaneous melanoma with tumors in the head and neck region between 2004 and 2021 at the Mayo Clinic and University Hospitals Cleveland Medical Center. Each institution followed its own guidelines for detecting nodal metastases during the study period, which may have influenced the reported SLN positivity rates across sites. The patients with node-positive disease received adjuvant therapy in accordance with the national clinical guidelines in effect at the time of diagnosis. None of the high-risk node-negative patients received adjuvant therapy.

All the patients were referred for an SLNB based on national clinical guidelines (SLNB-eligible) and underwent an SLNB within 90 days after diagnosis to assess SLN biopsy status. The patients with clinically palpable nodes were excluded. All the included patients were selected from previously reported larger cohorts.^7,9,15^ Data analysis adhered to the American Joint Committee on Cancer (AJCC) eighth-edition staging system.^1^ This study was conducted with retrospective data from archived samples, but samples were prospectively collected for SLNB status.

### CP-GEP Model

The study measured CP-GEP as described previously.^7,8^ Briefly, CP-GEP integrates clinicopathologic features (patient age at diagnosis and Breslow thickness) with the expression levels of eight genes from the primary tumor: *ITGB3*, *PLAT*, *SERPINE2*, *GDF15*, *TGFBR1*, *LOXL4*, *CXCL8,* and *MLANA*, as well as housekeeping genes *RLP0* and *ACTB*.^7^ To measure the aggressiveness of the tumor, CP-GEP was developed because the genes in the model can predict the metastatic potential of the tumor. As such, the model gives insights on nodal metastasis risk as well as disease recurrence.^7,13^ The CP-GEP model has binary output labels: low risk and high risk for nodal metastases and disease recurrence.^7,8,13^

**Fig. 1:**
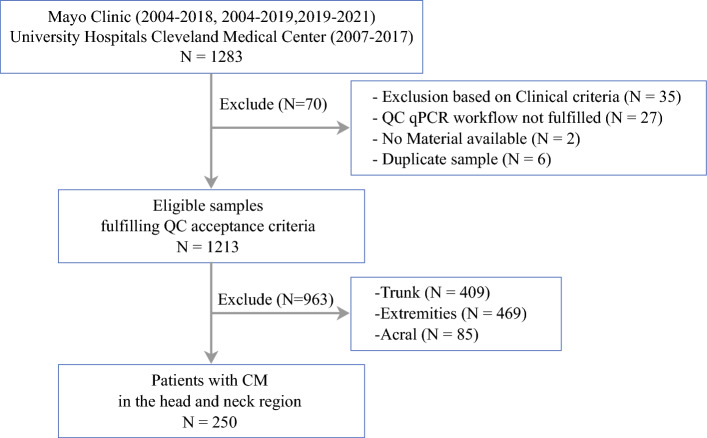
Consort diagram depicting the enrollment of patients and reasons for exclusion

From 1283 patient samples, the study excluded samples failing to meet necessary quality or quantity standards for gene expression profiling (*n *= 27), samples failing to meet clinical criteria (*n *= 35), duplicate samples (*n *= 6), samples for which no material was available (*n *= 2), and body locations other than the head and neck region (*n *= 963), leading to a final cohort of 250 patients with tumors in the head and neck region (consort diagram in Fig. 1).

Formalin-fixed paraffin-embedded (FFPE) blocks from each primary tumor were retrieved from the dermatopathology archives, and a total of 50 microns was used for the gene expression analysis. During CP-GEP analysis, SLNB status outcome was blinded for laboratory personnel. The CP-GEP model has a turnaround time of 5 working days, and the average failure rate is 5%.

### Statistical Methods: SLNB Prediction

We characterized the performance of the CP-GEP model by calculating its sensitivity, specificity, negative predictive value (NPV), positive predictive value (PPV), SLNB reduction rate (RR), and corresponding 95% Clopper-Pearson confidence intervals (CIs).^16^ The SLNB RR is defined as the proportion of patients classified as low risk by the model among all patients, calculated as previously described.^17^ We stratified all performance measures by T categories according to the AJCC eighth-edition staging system.^1^

Statistical analyses were performed using R software (4.2.2; R Foundation for Statistical Computing, Vienna, Austria),^18^ and *p* values lower than 0.05 were considered statistically significant. Patient characteristics were summarized using the gtsummary package in R (version 1.7.0).^19^

### Statistical Methods: Survival Outcomes

The prognostic ability of CP-GEP was evaluated using Kaplan-Meier curves. The clinical end points were recurrence-free survival (RFS), distant metastasis-free survival (DMFS), and melanoma-specific survival (MSS). The hazard ratio (HR) was calculated with a 95 % CI using a Cox proportional hazards regression model. A Wald *p* value lower than 0.05 (two-sided) was considered statistically significant. Follow-up evaluation was capped at 5 years. Patients experiencing an event beyond this duration were censored at the 5-year mark. The median follow-up period was determined using the reverse Kaplan-Meier estimator prodlim in R (version 2019.11.13).^20^

## Results

### Study Population

**Fig. 2:**
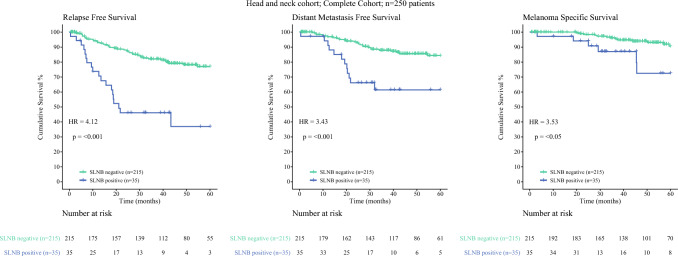
Kaplan-Meier analysis of the 250 patients with stages I to III melanoma in the head and neck region, stratified by sentinel lymph node biopsy (SLNB) outcome. The survival end points were relapse-free survival (RFS), distant metastasis-free survival (DMFS), and melanoma-specific survival (MSS) during the 5-year follow-up period. SLNB-negative (*light blue curve*); SLNB-positive (*dark blue curve*). For each of the end points, the hazard ratio (HR) and corresponding *p* value calculated with the Wald test are shown

The study enrolled 250 patients with primary cutaneous melanoma of the head and neck region. Most of the patients had stages I to IIA disease, totaling 76.4% of the entire cohort (*n *= 191). The median Breslow thickness was 1.6 mm (interquartile range [IQR], 1.1–2.5 mm), and 79.2% of the tumors were in male patients. The median age was 68 years (IQR, 56–76 years), and ulceration was absent in 79.6% of the tumors (Table 1). Nodal metastases were found in 35 patients, resulting in an SLNB positivity rate of 14%. The 5-year rates were 71.3% (95% CI, 64.2–77.3%) for RFS, 80.8% (95% CI, 74.2–85.8%) for DMFS, and 88.4% (95% CI, 82.1–92.7%) for MSS, with respective median follow-up periods of 48 months (IQR, 32–74 months), 46 months (IQR, 29–74 months) and 49 months (IQR, 33–80 months). The SLNB-positive patients had 5-year RFS of 36.8% (95% CI, 17.0–57.0%), DMFS of 61.4% (95% CI, 41.6–76.2%), and MSS of 72.5% (95% CI, 45.5–87.7%). The SLNB-negative patients had 5-year RFS of 76.9% (95% CI, 69.4–82.8%), DMFS of 84.3% (95% CI, 77.4–89.2%), and MSS of 90.6% (95% CI, 83.8–94.6%) (Fig. 2 and Table S1).

**Table 1:** Patient and tumor characteristics stratified by SLNB outcome for the entire cohort^a^

		SLNB positivity		
	All patients	Negative	Positive	
Characteristic	(*n* = 250) *n* (%)	(*n* = 215) *n* (%)	(*n* = 35) *n* (%)	*p* Value^a^
*Sex*				
Female	52 (20.8)	43 (20.0)	9 (25.7)	0.44
Male	198 (79.2)	172 (80.0)	26 (74.3)	
Median age: years (IQR)	68 (56–76)	68 (58–77)	58 (40–72)	0.007
Median Breslow thickness: mm (IQR)	1.60 (1.10–2.50)	1.50 (1.10–2.30)	2.30 (1.70–3.30)	< 0.001
*Biopsy location*				
Head neck	250 (100.0)	215 (100.0)	35 (100.0)	NA
*Histologic type*				
Superficial spreading	78 (31.2)	65 (30.2)	13 (37.1)	0.002
Nodular	50 (20.0)	40 (18.6)	10 (28.6)	
Desmoplastic	19 (7.6)	18 (8.4)	1 (2.9)	
Lentigo maligna	45 (18.0)	45 (20.9)	0 (0.0)	
Spindled	7 (2.8)	7 (3.3)	0 (0.0)	
Dermal	2 (0.8)	2 (0.9)	0 (0.0)	
Spitzoid	3 (1.2)	3 (1.4)	0 (0.0)	
Nevoid	7 (2.8)	6 (2.8)	1 (2.9)	
Unclassifiable	18 (7.2)	10 (4.7)	8 (22.9)	
Other	9 (3.6)	9 (4.2)	0 (0.0)	
Mixed	4 (1.6)	3 (1.4)	1 (2.9)	
Unknown	8 (3.2)	7 (3.3)	1 (2.9)	
*Clark level*				
II	1 (0.4)	1 (0.5)	0 (0.0)	< 0.001
III	29 (11.6)	29 (13.5)	0 (0.0)	
IV	141 (56.4)	114 (53.0)	27 (77.1)	
V	11 (4.4)	7 (3.3)	4 (11.4)	
Unknown	68 (27.2)	64 (29.8)	4 (11.4)	
*Ulceration*				
Absent	199 (79.6)	176 (81.9)	23 (65.7)	0.071
Present	49 (19.6)	37 (17.2)	12 (34.3)	
Unknown	2 (0.8)	2 (0.9)	0 (0.0)	
*Angiolymphatic invasion*				
Absent	164 (65.6)	140 (65.1)	24 (68.6)	< 0.001
Present	8 (3.2)	1 (0.5)	7 (20.0)	
Unknown	78 (31.2)	74 (34.4)	4 (11.4)	
*Mitotic rate*				
Absent	25 (10.0)	23 (10.7)	2 (5.7)	0.61
Present	224 (89.6)	191 (88.8)	33 (94.3)	
Unknown	1 (0.4)	1 (0.5)	0 (0.0)	
*T category*				
T1a	7 (2.8)	7 (3.3)	0 (0.0)	< 0.001
T1b	45 (18.0)	45 (20.9)	0 (0.0)	
T2a	98 (39.2)	88 (40.9)	10 (28.6)	
T2b	17 (6.8)	13 (6.0)	4 (11.4)	
T3	1 (0.4)	1 (0.5)	0 (0.0)	
T3a	51 (20.4)	38 (17.7)	13 (37.1)	
T3b	16 (6.4)	12 (5.6)	4 (11.4)	
T4a	7 (2.8)	7 (3.3)	0 (0.0)	
T4b	8 (3.2)	4 (1.9)	4 (11.4)	

SLNB, sentinel lymph node biopsy; IQR, interquartile range

^a^Categorical variables are reported as total numbers (%) and and continuous variables as medians (IQR)

### CP-GEP Performance for Predicting SLN Status and Long-Term Survival for Stages I to III Patients

**Fig. 3:**
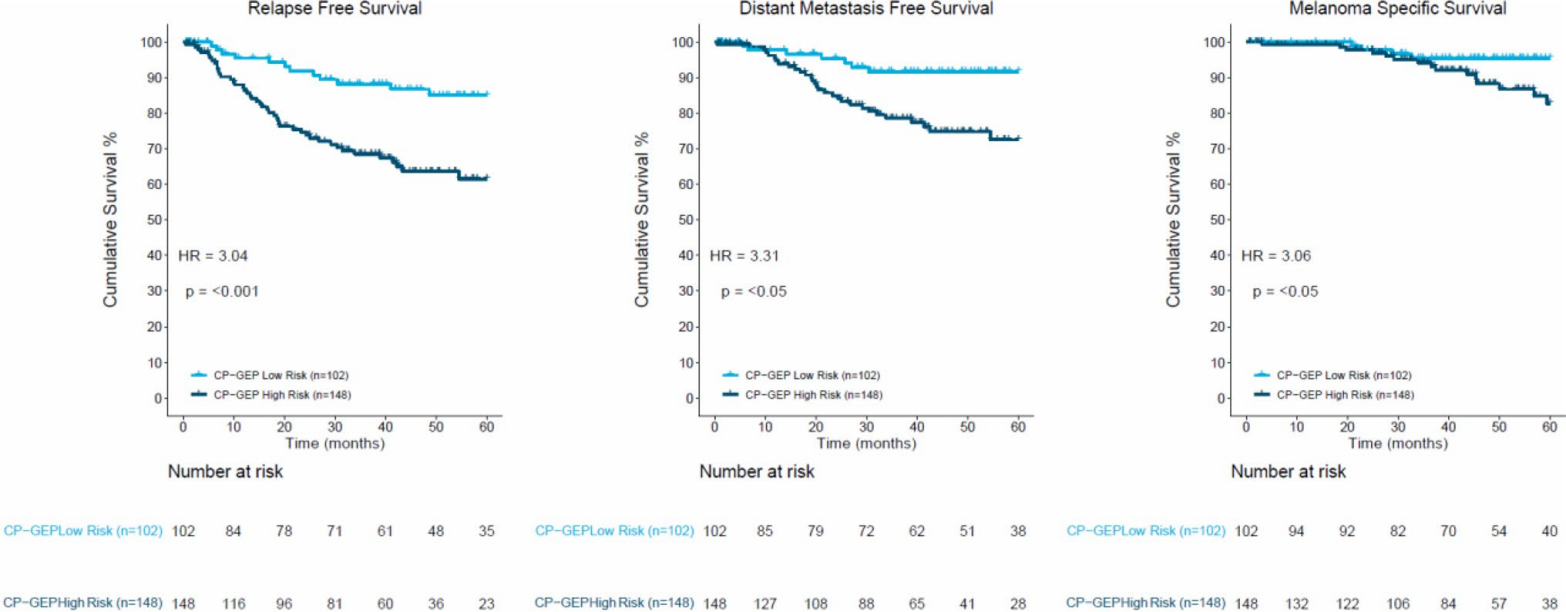
Kaplan-Meier analysis of the 250 patients with stage I, II, or III melanoma in the head and neck region, stratified by CP-GEP classification. The survival end points were relapse-free survival (RFS), distant metastasis-free survival (DMFS), and melanoma-specific survival (MSS) during the 5-year follow-up period. CP-GEP low risk (*light blue curve*); CP-GEP high risk (*dark blue curve*). For each of the end points, the hazard ratio (HR) and corresponding *p* value calculated with the Wald test are shown. CP-GEP, a model that combines clinicopathologic and gene expression variables

In this cohort of 250 patients, CP-GEP classified 102 patients as low risk and 148 patients as high risk. The NPV of CP-GEP for SLNB status was 98.0% (95% CI, 93.1–99.8%) (i.e., 100 of 102 patients with a low-risk label were indeed SLNB-negative). The CP-GEP model achieved an SLNB RR of 40.8% (95% CI, 34.6–47.2%; Table 2). When stratified by CP-GEP, the 5-year RFS rate was 85% (95% CI, 75.0–91.3%) for the low-risk patients versus 61.4% (95% CI, 51.2–70.2%) for the high-risk patients (hazard ratio [HR], 3.04; *p* < 0.001; Fig. 3). The low-risk patients had 5-year DMFS of 91.6% (95% CI, 83.1–95.9%) and MSS of 95.4% (95% CI, 88.2–98.3%) versus 5-year DMFS of 72.6% (95% CI, 62.4–80.4%) and MSS of 82.6% (95% CI, 71.8–89.5%) for the high-risk patients (Fig. 3; Table S1). Among 167 T1–T2 patients, the SLNB positivity rate was 8.4% (95% CI, 4.7–13.7%), and CP-GEP achieved an SLNB RR of 56.3% (95% CI, 48.4–63.9%) with an NPV of 98.9% (95% CI, 94.2–100%) (Table 2).

**Table 2:** T category performance of CP-GEP on entire cohort^a^

Patient subset	*n*	SLNB positivity rate (95% CI)	Specificity (95% CI)	Sensitivity (95% CI)	PPV (95% CI)	NPV (95% CI)	TP	TN	FP	FN	SLNB RR (95% CI)
T1–T2	167	8.4 (4.7–13.7)	60.8 (52.6–68.6)	92.9 (66.1–99.8)	17.8 (9.8–28.5)	98.9 (94.2–100)	13	93	60	1	56.3 (48.4–63.9)
T1–T3	235	13.2 (9.1–18.2)	48.5 (41.5–55.6)	93.5 (78.6–99.2)	21.6 (15–29.6)	98 (93–99.8)	29	99	105	2	43 (36.6–49.6)
T1–T4	250	14 (9.9–18.9)	46.5 (39.7–53.4)	94.3 (80.8–99.3)	22.3 (15.9–29.9)	98 (93.1–99.8)	33	100	115	2	40.8 (34.6–47.2)
T1	52	0 (0–6.8)	73.1 (59–84.4)	–	0 (0–23.2)	100 (90.7–100)	0	38	14	0	73.1 (59–84.4)
T2	115	12.2 (6.8–19.6)	54.5 (44.2–64.4)	92.9 (66.1–99.8)	22 (12.3–34.7)	98.2 (90.4–100)	13	55	46	1	48.7 (39.3–58.2)
T3	68	25 (15.3–37)	11.8 (4.4–23.9)	94.1 (71.3–99.9)	26.2 (15.8–39.1)	85.7 (42.1–99.6)	16	6	45	1	10.3 (4.2–20.1)
T4	15	26.7 (7.8–55.1)	9.1 (0.2–41.3)	100 (39.8–100)	28.6 (8.4–58.1)	100 (2.5–100)	4	1	10	0	6.7 (0.2–31.9)

CP-GEP, a model that combines clinicopathologic and gene expression variables; SLNB, sentinel lymph node biopsy; PPV, positive predictive value; NPV, negative predictive value; TP, true positive; TN, true negative; FP, false-positive; FN, false-negative; RR, reduction rate

^a^Performance was characterized by calculating sensitivity, specificity, NPV, PPV, SLNB, RR, and corresponding 95 % Clopper-Pearson confidence interval

### CP-GEP Performance for Predicting SLN Status and Long-Term Survival for Stages I and II Patients

**Fig. 4:**
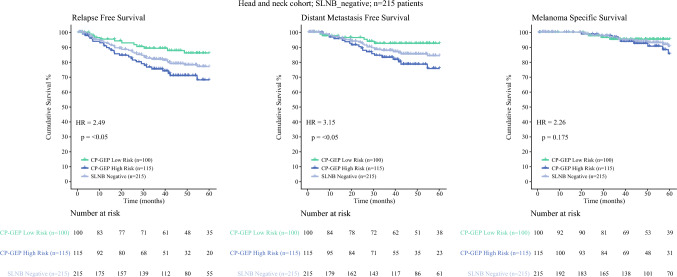
Kaplan-Meier analysis of the 215 sentinel lymph node biopsy (SLNB)-negative patients with stage I or II melanoma in the head and neck region, stratified by CP-GEP classification. The survival end points were relapse-free survival (RFS), distant metastasis-free survival (DMFS), and melanoma-specific survival (MSS) during the 5-year follow-up period. CP-GEP low risk (*light blue curve*); CP-GEP high risk (*dark blue curve*); total group of SLNB-negative patients (*gray curve*). For each of the end points, the hazard ratio (HR) and corresponding *p* value calculated with the Wald test are shown. CP-GEP, a model that combines clinicopathologic and gene expression variables

For 215 SLNB-negative patients, CP-GEP classified 100 patients as low risk and 115 patients as high risk. The 5-year RFS rate was 86.1% (95% CI: 76.1–92.1%) for the low-risk patients versus 68.1% (95% CI, 56.1–77.5%) for the high-risk patients (HR, 2.49; *p* < 0.05; Fig. 4). For the other clinical end points, DMFS and MSS, the 5-year survival rates were respectively 92.7% (95% CI, 84.5–96.7%) and 95.3% (95% CI, 88–98.2%) for the low-risk patients versus 75.8% (95% CI, 63.8–84.3%) and 85.5% (95% CI, 72.9–92.6%) for the high-risk patients (Fig. 4; Table S2).

## Discussion

The National Comprehensive Cancer Network (NCCN) guidelines recommend SLNB for melanomas staged T1a with adverse features, T1b, or higher.^21^ However, head and neck melanomas pose unique diagnostic and treatment challenges compared with melanomas located in other body regions.^6^

Identifying true sentinel lymph nodes in the head and neck region is challenging due to the complex lymphatic network and often tiny size of the individual lymph nodes in this area.^4,5^ Ongoing research is focused on developing more effective methods for risk assessment and patient monitoring. The Merlin Assay (CP-GEP) addresses this need by enhancing risk stratification beyond traditional methods.^7^

In this study we found that long-term survival outcomes were significantly better for the CP-GEP low-risk patients than for the CP-GEP high-risk patients across all survival end points (85.0%, 91.6%, 95.4% vs 61.4%, 72.6%, 82.6% for RFS, DMFS, and MSS, respectively). Among the SLNB-negative patients, CP-GEP was able to further categorize these early-stage melanoma patients according to their risk of recurrence by identifying 100 patients as low risk with 5-year RFS, DMFS and MSS rates of 86.1%, 92.7%, and 95.3% compared with 76.9%, 84.3%, and 90.6%, respectively, for the SLNB-negative patients. This illustrates that traditional staging with SLNB can be improved by the Merlin Assay.

Conversely, a positive SLNB result indicated a very high likelihood of relapse (RFS of 36.8%; MSS of 72.5%), underscoring the importance of not overlooking patients truly at risk for nodal metastasis who should undergo SLNB. It was therefore encouraging to find that the CP-GEP model produced very few false-negative results (i.e., results inaccurately predicting non-metastatic SLNs when the nodes were positive) with an NPV of 98%.

Analysis of our cohort showed that most melanomas in the head and neck region are present in males (79.2%), and that 46% of patients have T2 melanomas, reflected by relatively low 5-year survival rates of 71.3% for RFS, 80.8% for DMFS, and 88.4% for MSS in the complete cohort. For the T1–T2 patients, SLNB reduction rates reaching 56.3% were achieved with an NPV of 98.9%, illustrating the proportion of patients who could have safely avoided SLNB based on the CP-GEP model’s prediction of a low risk for nodal metastases.

This study had several key strengths. First, its study design, which prospectively collected data on archived samples for SLNB status, enhanced the reliability of its findings and minimizes bias. Second, the multicenter nature of the study enhanced the generalizability of the results and reduced institutional bias. Third, this study had a substantial sample size of tumors in the head and neck region (250 patients), increasing the power of our statistical analysis.

We believe that implementing the CP-GEP model in clinical practice will not delay SLNB surgeries because the turnaround time for CP-GEP testing is 5 working days, well within the typical referral-to-consultation time frame for SLNB (2–3 weeks).

Regarding study limitations, our sample may have been too small to evaluate the impact of potential clinical biases, such as the over- or under-representation of specific clinical characteristics such as age, sex, and histologic subtype. Furthermore, our follow-up period was truncated, at 5 years, which may have failed to capture long-term outcomes and recurrence patterns beyond this time frame, potentially leading to an underestimation of the impact of certain interventions. Additionally, although the location of recurrences, such as lung or other organs, could have offered valuable insights into the specificity of the gene profile for metastasis to certain sites, we did not have access to these data for our study cohort and were unable to investigate these details.

In conclusion, these data underscore the utility of the Merlin Assay for managing primary cutaneous melanoma in patients with tumors in the head and neck region. This subset of melanoma patients faces heightened challenges with SLNB and a higher SLN false-negative rate than those with tumors in other body locations. Implementation of the Merlin Assay holds promise to enhance clinical management by improving patient selection for SLNB while also giving insights on long-term survival outcome, thereby improving the precision of melanoma patient care and optimizing health care resources.

## Supplementary Information

Below is the link to the electronic supplementary material.

Supplementary file1 (DOCX 13 KB)
