# Correction: Enhanced Risk Stratification for Sentinel Lymph Node Biopsy in Head and Neck Melanoma Using the Merlin Assay (CP-GEP)

**DOI:** 10.1245/s10434-025-16946-1

**Published:** 2025-01-30

**Authors:** Ani Pazhava, Wesley Y. Yu, Frank Z. Jing, Sheena Hill, Bethany R. Rohr, Kord S. Honda, Félicia Tjien-Fooh, Renske Wever, Jvalini Dwarkasing, Tina J. Hieken, Alexander Meves

**Affiliations:** 1 Department of Dermatology, Mayo Clinic, Rochester, MN USA; 2 Department of Dermatology, Oregon Health and Science University, Portland, OR USA; 3 Department of Dermatology, University Hospitals Cleveland Medical Center, Cleveland, OH USA; 4 SkylineDx B.V., Rotterdam, The Netherlands; 5 Department of Surgery, Mayo Clinic, Rochester, MN USA

**Correction to: Annals of Surgical Oncology** 10.1245/s10434-024-16551-8

In the original online version of this article there was an error in Fig. 4. The dark blue line was incorrectly indicated as CP-GEP Low Risk. It is CP-GEP High Risk.

The original article was corrected.

