# Correction: Enhanced Risk Stratification for Sentinel Lymph Node Biopsy in Head and Neck Melanoma Using the Merlin Assay (CP-GEP)

**DOI:** 10.1245/s10434-025-17805-9

**Published:** 2025-07-12

**Authors:** Ani Pazhava, Wesley Y. Yu, Frank Z. Jing, Sheena Hill, Bethany R. Rohr, Kord S. Honda, Félicia Tjien-Fooh, Renske Wever, Jvalini Dwarkasing, Tina J. Hieken, Alexander Meves

**Affiliations:** 1 Department of Dermatology, Mayo Clinic, Rochester, MN USA; 2 Department of Dermatology, Oregon Health and Science University, Portland, OR USA; 3 Department of Dermatology, University Hospitals Cleveland Medical Center, Cleveland, OH USA; 4 SkylineDx B.V., Rotterdam, The Netherlands; 5 Department of Surgery, Mayo Clinic, Rochester, MN USA

**Correction to: Ann Surg Oncol (2025) 32:2748–2755** 10.1245/s10434-024-16551-8

In the original online version of this article, the HR and *p* values in Fig. 3 were incorrect.

The article “ Enhanced Risk Stratifcation for Sentinel Lymph Node Biopsy in Head and Neck Melanoma Using the Merlin Assay (CP–GEP)“, written by Pazhava et al., was originally published electronically on the publisher’s internet portal on November 23, 2024, without open access. With the authors’ decision to opt for Open Choice the copyright of the article changed on July 3, 2025 to © The Author(s) 2024 and this article is licensed under a Creative Commons Attribution 4.0 International License, which permits use, sharing, adaptation, distribution and reproduction in any medium or format, as long as you give appropriate credit to the original author(s) and the source, provide a link to the Creative Commons licence, and indicate if changes were made. The images or other third party material in this article are included in the article’s Creative Commons licence, unless indicated otherwise in a credit line to the material. If material is not included in the article’s Creative Commons licence and your intended use is not permitted by statutory regulation or exceeds the permitted use, you will need to obtain permission directly from the copyright holder. To view a copy of this licence, visit http://creativecommons.org/licenses/by/4.0/.

The original article was corrected.

