# Supplementary material for: Enhanced Risk Stratification for Sentinel Lymph Node Biopsy in Head and Neck Melanoma Using the Merlin Assay (CP-GEP)
Source: Ann Surg Oncol. 2024 Nov 23;32(4):2748–55. doi: 10.1245/s10434-024-16551-8 (PMC12255597; doi:10.1245/s10434-024-16551-8)
Supplement: Supplementary file 1 — Supplementary file1 (DOCX 13 KB) [file 10434_2024_16551_MOESM1_ESM.docx]

**Supplementary table 1.** Survival endpoints at five years of follow-up for complete cohort: relapse-free survival (RFS), distant metastasis-free survival (DMFS) and melanoma-specific survival (MSS).

|  | **N** | **Events**  **RFS** | **RFS (%),**  **95%CI** | **Events**  **DMFS** | **DMFS (%),**  **95%CI** | **Events**  **MSS** | **MSS (%),**  **95%CI** |
| --- | --- | --- | --- | --- | --- | --- | --- |
| **Complete Cohort (Stage I-III)** | 250 | 57 | 71.3 [64.2-77.3] | 37 | 80.8 [74.2-85.8] | 19 | 88.4 [82.1-92.7] |
| SLNB negative | 215 | 38 | 76.9 [69.4-82.8] | 25 | 84.3 [77.4-89.2] | 13 | 90.6 [83.8-94.6] |
| SLNB positive | 35 | 19 | 36.8 [17-57] | 12 | 61.4 [41.6-76.2] | 6 | 72.5 [45.5-87.7] |
| CP-GEP Low Risk | 102 | 12 | 85.0 [75-91.3] | 7 | 91.6 [83.1-95.9] | 4 | 95.4 [88.2-98.3] |
| CP-GEP High Risk | 148 | 45 | 61.4 [51.2-70.2] | 30 | 72.6 [62.4-80.4] | 15 | 82.6 [71.8-89.5] |

|  | **N** | **Events**  **RFS** | **RFS (%),**  **95%CI** | **Events**  **DMFS** | **DMFS (%),**  **95%CI** | **Events**  **MSS** | **MSS (%),**  **95%CI** |
| --- | --- | --- | --- | --- | --- | --- | --- |
| **SLNB negative (stage I-II)** | 215 | 38 | 76.9 [69.4-82.8] | 25 | 84.3 [77.4-89.2] | 13 | 90.6 [83.8-94.6] |
| CP-GEP Low Risk | 100 | 11 | 86.1 [76.1-92.1] | 6 | 92.7 [84.5-96.7] | 4 | 95.3 [88-98.2] |
| CP-GEP High Risk | 115 | 27 | 68.1 [56.1-77.5] | 19 | 75.8 [63.8-84.3] | 9 | 85.5 [72.9-92.6] |

**Supplementary table 2.** Survival endpoints at five years of follow-up for SLNB negative (stage I-II) subgroup: relapse-free survival (RFS), distant metastasis-free survival (DMFS) and melanoma-specific survival (MSS).
